# Strategies for advanced personalized tuberculosis diagnosis: Current technologies and clinical approaches

**DOI:** 10.1093/pcmedi/pbaa041

**Published:** 2021-01-18

**Authors:** Xuerong Chen, Tony Y Hu

**Affiliations:** Department of Respiratory and Critical Care Medicine, West China Hospital, Sichuan University, Chengdu 610041, China; Center for Cellular and Molecular Diagnostics, School of Medicine, Tulane University, New Orleans, LA 70112, USA

**Keywords:** tuberculosis, molecular diagnosis, personalized medicine

## Abstract

Diagnosis of tuberculosis can be difficult as advances in molecular diagnosis approaches (especially nanoparticles combined with high-throughput mass spectrometry for detecting mycobacteria peptide) and personalized medicine result in many changes to the diagnostic framework. This review will address issues concerning novel technologies from bench to bed and new strategies for personalized tuberculosis diagnosis.

## Introduction

Mycobacterium tuberculosis (MTB) is the most infectious single pathogen in the world, causing infection in an estimated 10.0 million people in 2019 globally, and leading to 1.4 million deaths in 2019. Close to half a million people developed rifampicin-resistant TB (RR-TB), of whom 78% had multidrug-resistant tuberculosis (MDR-TB). The TB incidence rate is falling, but not fast enough to reach the 2020 milestone of a 20% reduction between 2015 and 2020. The cumulative reduction from 2015 to 2019 was 9%. The COVID-19 pandemic threatens to reverse recent progress in reducing the global burden of TB disease as a result of decreased Gross Domestic Product (GDP) per capita and undernutrition. It is estimated that the number of people developing TB could increase by more than 1 million per year in the period 2020–2025 by constructing models.^1^

Because of underreporting of detected cases and underdiagnosis, there is still a wide gap (2.9 million patients) between the verified and evaluated cases of TB. Furthermore, only one-third of patients with MDR-TB/RR-TB received antibiotic treatment.^1^ To decrease the global prevalence of TB, it is critical to improve the level of case-finding and diagnosis. Increasing case-finding can be accomplished using two approaches: a patient-initiated approach, in which patients voluntarily visit the doctor when they have common TB symptoms such as cough, weight loss, night sweats, and fever; and an active case-finding approach, in which patients are tested if they have an increased chance of contracting TB, which includes close contact with TB-positive patients and HIV-infected individuals. TB has the nickname “universal imitator” and can be confused with carcinoma, fungal disease and autoimmune disease, and other diseases. Therefore, doctors need to be alert to differentiate TB from other diseases, especially in countries with high TB burden.

**Figure 1.**
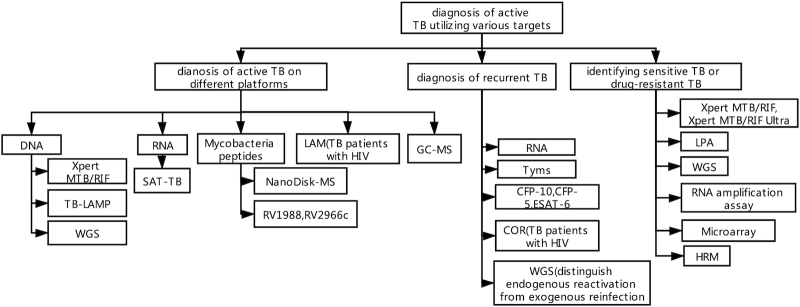
Diagnosis of active TB utilizing various targets.

We focus not only on the diagnosis of TB itself but also on the diagnosis of drug resistance. In addition to sputum smear microscopy for acid-fast stain, mycobacterial culture, chest X-ray, WHO-endorsed Xpert MTB/RIF, line probe assay (LPA), and loop-mediated isothermal amplification (LAMP) are new diagnostic methods (Fig. 1, Table 1). In 2017, 32 countries adopted Xpert MTB/RIF as the WHO-recommended rapid diagnostic (WRD) test for detection of mycobacteria tuberculosis (MTB)-DNA and rifampin resistance.^1^ Further improvements in TB diagnostics need to include:

- More sensitive, cost-effective diagnostic tools for rapid detection of MTB and drug-sensitive test results;
- Accurate information to differentiate between active, latent, subclinical, and old TB;
- Novel biomarkers to monitor therapy outcomes.

**Table 1.** Sensitivities and specificities of PTB diagnostic tests.

Tool (sample)	Sensitivity*	Specificity*	Advantages and defeats	Price
Xpert (sputum)	65%–90%^2,3^	>98%^2,3^	High specificity; and fluctuate sensitivities	$85
TB-LAMP (sputum)	65%–84.4%^3,8,10^	90%–98.7%^3,8,10^	Cheap; need simple instrumentation	$36
WGS (isolates)	95%^12^	98%^12^	Accurate; expensive; need experts and complex instrumentation	$481
SAT (sputum)	75.8%–96%^15,16^	88%–100%^15,16^	Cheap; can detect live mycobacteria	$30
NanoDisk-MS (blood)	91.6%–92.3%^18,19^	95.8%^18,19^	Accurate; do not need to handle infectious samples; can detect live mycobacteria	NA
LAM (urine)	39%–66.7%^22,23^	>98%^22,23^	Cheap; need simple instrumentation; high sensitivity in the HIV(+) subjects	$3.5
GC-MS (breath)	62%^24^	84%^24^	Noninvasive; low sensitivity and specificity	NA

TB-LAMP: tuberculosis-loop-mediated isothermal amplification; WGS: whole-genome sequencing; SAT: simultaneous amplification and testing; LAM: lipoarabinomannan; GC-MS: gas chromatography-mass spectrometry.

* The gold standard for confirming TB is sputum culture.

## Diagnosis of active TB utilizing various targets

### Diagnosis of active TB on different platforms

#### Diagnosis based on the detection of mycobacteria DNA

The Xpert MTB/RIF Assay (Cepheid, Sunnyvale, CA, USA) is a widely used, automated molecular method based on nested PCR. The sensitivity was indicated to be 78.2%–90% in patients with culture-positive pulmonary tuberculosis (PTB), and the specificity was >98%. In children, the sensitivity decreased to 65%–76%.^2,3^ In addition to sputum, other specimens, such as induced sputum, gastric aspiration, and stool, have also been collected for the detection of TB. A meta-analysis showed that when compared to microbiological reference standards, the pooled sensitivity and specificity of stool Xpert results among children were 67% and 99%, respectively, and the sensitivity was higher among children with HIV (79% and 60%, respectively, among HIV-infected and HIV-uninfected children), suggesting that stool Xpert is fit for diagnosing PTB in children, particularly those with HIV.^5^

Technical innovations have been made continuously for TB diagnosis. A portable type diagnostic tool, Xpert Omni, is being produced to offer a more flexible way to examine patients.^6^ Furthermore, a new version of this test, Xpert Ultra, has a better sensitivity and slightly lower specificity than Xpert, and false-positive Xpert Ultra results were usually found in adults previously treated for TB. Therefore, Xpert Ultra has the potential to increase the reliability and efficiency of diagnosing tuberculosis in children in areas with a high burden of tuberculosis and HIV. The induced sputum Xpert MTB/RIF Ultra assay was applied to diagnose pulmonary tuberculosis in South African children with a sensitivity of 75.3% and a specificity of 96.9%.^7^

TB-LAMP is a novel, rapid approach for nucleic acid detection based on loop-mediated isothermal amplification technology. It takes less than 2 h to perform, and the results can be determined by the naked eye. This test was recommended by the WHO as an alternative to microscopy in adults and an additional test to microscopy after Ziehl-Neelsen stain was negative for the smear of the sputum. In two multicenter investigations, TB-LAMP accurately detected TB with a total sensitivity of 75.6%–84.4%, which included sensitivities of 97.2%–97.9% in smear-positive TB samples and 46.6%–62% in smear-negative TB samples, and a specificity of 96.6%–98.7%.^3,8^ Our study suggested sensitivity of 87.5% and specificity of 89.3% in a suspected TB group with 80 subjects from West China area, using culture as gold standard. The results varied widely by country and operator. High temperatures, high humidity, and/or low reaction volumes were identified as possible causes for false-positive results because of nonspecific amplification. The diagnostic value of TB-LAMP is comparable with that of Xpert, and complicated infrastructure is not needed; therefore, this test is suitable for decentralized settings. A meta-analysis comparing TB-LAMP and sputum smears suggested that utilizing TB-LAMP for diagnosis has a higher sensitivity (66%–91%) than that in smear microscopy and a similar sensitivity to that in Xpert MTB/RIF using a culture standard. In addition, the specificity (90%–98%) of TB-LAMP was similar to those of smear microscopy and Xpert MTB/RIF. As an additional test in smear-negative patients, TB-LAMP had fluctuating sensitivities (19%–78%).^9^ However, not all the results were optimal. A study conducted in Malawi indicated that the sensitivity (65.0%) of TB-LAMP was similar to that of Xpert (77.5%) but was lower than that of fluorescence smear microscopy (87.5%). Moreover, TB-LAMP lacks a cost advantage over fluorescence smear microscopy.^10^ The diagnostic value for expulmonary tuberculosis (EPTB) was also assessed. A meta-analysis observed pooled sensitivity of 77% and specificity of 99% of TB-LAMP for the detection of EPTB, respectively, when compared to a composite reference standard (composed of clinical symptoms, biochemical testing results, bacteriological results, histopathology, other nucleic acid amplification tests, or responses to anti-tuberculosis treatment), and sensitivity of 93% and specificity of 77%, respectively, when compared to cultures alone. The controversial conclusion might be explained by the different target genes used in the included studies and the heterogeneity of different studies.^11^

Whole genome sequencing (WGS) is a rapid and precise approach to differentiate TB from other diseases. In a prospective study, mycobacterium tuberculosis (MTB) was identified in 345 BACTEC MGIT culture isolates with a sensitivity of 95% and a specificity of 98%.^12^ This test is not only used for culture isolates but also for sputa directly.^13^ An Illumina MiniSeq sequencer is used to deliver same-day test results (16 h) with a relatively low cost ($198). Another instrument, MinION (miniaturized sequencing platform), allows sequencing to continue until enough coverage has been obtained.^14^ It can also detect the genomes of other pathogens, including viruses, bacteria, and fungi, so some mixed infections that usually occur in immunocompromised patients can be diagnosed easily. Targeted NGS (next generation sequence) could be employed as a rapid diagnostic tool because of its cheap price and lack of need for culture.

In summary, WGS is an accurate and efficient tool for diagnosing TB, but two obstacles need to be overcome for its use: i) isolation of genomes of mycobacteria from human counterparts; and ii) exploration of more powerful bioinformatics tools to analyze data and further combine the clinical data.

#### Diagnosis based on detection of mycobacterial RNA

A large cohort study (3608 patients) revealed that the sensitivity, specificity, and accuracy of the simultaneous amplification and testing (SAT-TB) method were 75.8%, 100%, and 80.2%, respectively, for diagnosis of PTB.^15^ A meta-analysis revealed pooled sensitivity and specificity (using sputum samples) for the diagnosis of PTB of 96% and 88% for SAT, 93% and 94% for TB-LAMP, and 89% and 98% for Xpert, respectively. SAT is more sensitive than TB-LAMP and Xpert for the detection of smear-negative PTB. It is suitable for use for optimal and reimbursable cost.^16^ However, the sensitivity and specificity of the SAT-TB assay declined to 51% and 95%, respectively, when SAT was applied using bronchoalveolar lavage fluid (BALF) samples for detecting sputum-scarce PTB.^17^

#### Diagnosis based on detection of mycobacterial peptides

Hu *et al*. utilized antibody-labeled and energy-focusing porous discoidal silicon nanoparticles (NanoDisk) and high-throughput mass spectrometry (MS) to rapidly detect the serum concentrations of antigens. The sensitivities for culture-negative PTB and EPTB in HIV-positive cases were 91.3% and 92.3%, respectively.^18^ In another study, NanoDisk-MS also diagnosed patients with paucibacillary TB disease with a specificity of 95.8% and sensitivities of 91.6% and 85.3% for culture-positive and -negative TB cases, respectively.^19^

Some novel peptides have promising diagnostic value. Rv1988, a secretory protein of MTB, dimethylates the R42 residue of histone H3 and decreases the expression of host genes that are involved in the host defense against mycobacteria.^20^ Another secretory protein, Rv2966c has a similar mechanism to regulate host genes. It is a mycobacterial DNA methyltransferase that might induce non-CpG DNA methylation, located in a few gene families involved in the immune response and in chromatin reorganization in the host macrophage during an infection with MTB.^21^ These peptides are virulent factors that subvert the host immune system and could hopefully be detected in the blood of the host.

#### Diagnosis based on detection of mycobacterial lipoarabinomannan (LAM)

LAM is a major constituent of the MTB cell wall. Its presence in the urine of the host indicates activation of MTB and can be tested by ELISA. However, the sensitivities of this test vary (14% vs. 51%) between active TB patients with and without HIV infection,^22^ and the sensitivity decreases gradually depending on the increase in the CD4^+^ count in the blood (39.0% with a CD4 count of <200 cells/μL, 51.7% with a CD4 count of <100 cells/μL, and 66.7% with a CD4 count of <50 cells/μL).^23^ This method is more significant in patients with a TB-HIV coinfection, so it is suitable for such a population in detection of trace levels of TB.

#### Diagnosis based on detection of host metabolites

Host metabolites are small molecular compounds in host cells that participate in metabolic processes for normal cellular work. They can reflect the interaction between the host and microbe, and might have the potential for diagnosing TB or differentiating TB from other diseases. Some host metabolites have been identified by omics technologies and mined by bioinformatics approaches. Host metabolites can be obtained from different sample sources, which include blood, tissues, urine, and breath. Sputum has seldom been used because of its viscosity and uneven consistency. Kolk *et al*.^24^ built a classification model for predicting TB, and seven biosignatures were identified from breath samples. This model had a sensitivity of 62% and a specificity of 84% in the validated test. Some abnormal host fatty acid and amino acid metabolites were highlighted. By using liquid chromatography-mass spectrometry (LC-MS), Zhong *et al*.^25^ identified 61 biomarkers related to glycerophospholipid and arachidonic acid metabolism in the blood of TB-positive patients compared to those from healthy controls. Although the authors only explored their underlying mechanism for pathogenesis, these molecules still have significance in diagnosis of TB. Luies and Loots^26^ identified 12 urinary TB biomarkers in active TB patients by time-of-flight mass spectrometry (TOFMS). Most of the abnormalities involved tryptophan, phenylalanine, and tyrosine metabolism, which were probably mediated by IFN-γ and insulin.

This approach is advanced, and some technologies are invasive. In addition, the concentrations of the biomarkers are small, and a single compound specific to TB was not detected. Moreover, discrepancies exist in the concentrations of many metabolite markers.

### Diagnosis of recurrent TB

The existence of protein and RNA of mycobacteria strongly suggests the activity of TB. NanoDisk-MS is a promising method for protein determination. It detected serum CFP-10 levels in patients with recurrent TB with a sensitivity of 77.8% (40.0%–97.2%), which was equal to that in the culture technique.^27^ However, large cohort studies in different populations are needed for validation. Another two antigens have been specified as candidate antigens, ESAT-6 or CFP-5. Besides this, Darboe *et al*. developed a whole blood transcriptomic correlate of risk (COR) signature concerning 11 genes to predict recurrent tuberculosis in HIV-infected persons, with specificity of 75% and sensitivity of 60% within 3 months of clinical diagnosis.^28^ It is well known that examining DNA is not helpful when we address recurrent TB. WGS could be used to differentiate recurrent TB originating from either endogenous reactivation or exogenous reinfection by comparing genomes of the strains isolated with the primary counterparts. Thus, WGS has benefits in tracing potential TB spreaders and identifying transmission of the disease.^29^

### Identifying sensitive TB or drug-resistant TB

Rapid diagnostic assays for evaluating the drug sensitivity of mycobacteria are listed in Table 2. Xpert can detect the existence of rifampicin resistance through detection of the rpoB gene. The sensitivity of testing rifampicin resistance is 95%. Xpert MTB/RIF Ultra could improve the accuracy of detection of rifampicin resistance.^30^ However, determination of rifampicin resistance only is not sufficient for clinical doctors when they decide a regimen, as some patients with other drug-resistant TB would be missed. A new cartridge is being exploited for detecting resistance to isoniazid and other second-line drugs lacking in the original cartridge. The sensitivities were 83.3% for isoniazid, 88.4% for ofloxacin, 96.2% for moxifloxacin, 71.4% for kanamycin, and 70.7% for amikacin, and the specificities were 94.3% and above for all drugs except moxifloxacin, which had a specificity of 84.0%.^31^

**Table 2.** Sensitivities and specificities of tests for identifying drug-resistant PTB.

Method	Sensitivity	Specificity	Endorsed by the WHO?
Xpert	95% (RFP)^30,31^	94.3% (RFP\INH\Km)^30,31^	Yes
		84% (Mfx)	
	83.3% (INH)		
	96.2% (Mfx)		
	96.2% (Km)		
LPA	100% (RFP)^32–35^	98% (RFP)^32–35^	Yes
	92% (INH)	99% (INH)	
	80.5% (FQNs)	100% (FQNs)	
	80.7% (Km)	99.3% (Km)	
WGS	98% (RFP)^36,37^	98% (RFP)^36,37^	No
	97% (INH)	93% (INH)	
RNA amplification assay	100% (RFP)^38^	97.3% (RFP)^38^	No
Microarray	86.1% (RFP)^39^	97.7% (RFP)^39^	No
	79.4% (INH)	98.7% (INH)	
HRM	89%–94% (RFP)^40,41^	99%–100%	No
	85% (INH-KatG)	(RFP)^40,41^	
	100% (INH-InhA)	100% (INH)	

LPA: line probe assay; WGS: whole-genome sequencing; RNA: ribonucleic acid; HRM: high resolution melting; RFP: rifampicin; INH: isoniazid; Km: kanamycin; Mfx: moxifloxacin; FQNs: fluoroquinolone.

.

LPA is another molecular method based on nucleic acid amplification by PCR and detection of gene mutations by membrane-bound probes, and is available for first- and second-line drugs. It was reported that the test was able to detect mutations in the rpoB gene in MTB with a specificity and sensitivity of 98% and 100%, respectively, in India, and was popularized in TB high-burden countries because it is inexpensive.^32^ When used to judge the resistance of isoniazid (INH), the specificity and sensitivity were 92% and 99%, respectively.^33^ Desikan *et al*. found that of the 1217 isolates from patients suspected of having MDR-TB, 232 (19.1%) were MDR, 130 (10.6%) were rifampicin-monoresistant, and 101 (8.3%) were isoniazid-monoresistant.^34^ In a study of 353 MDR-TB specimens, the sensitivities for the detection of the resistance to fluoroquinolones, second-line injectable drugs, and extensive drug-resistance (XDR) were 80.5%, 80.7%, and 73.5%, respectively, and the specificities were 100.0%, 99.3%, and 99.1%, respectively.^35^

WGS, which can be utilized to determine if mycobacteria are drug-resistant, is an additional technique to determine resistance. This test could ideally detect all the mutations with their functional categorization but not part of them in the specified locus. Advantages for WGS are that false-negative and false-positive tests for resistance are avoided because of presentation of an unbiased view of the whole genome, so an optimal treatment regimen could be implemented without delay. Besides, WGS can identify the resistance determining mutations in new drugs. A meta-analysis suggested that in 23 studies, polymorphisms in 53 genes were tested for having an association with drug resistance. Pooled sensitivity and specificity values for the detection of resistance were 98% and 98% for rifampicin and 97% and 93% for isoniazid, respectively.^36^ A prospective study of 672 specimens predicted drug susceptibility (including first- and second-line drugs) with 93% accuracy.^12^ The most common mutations of isoniazid, rifampicin, ofloxacin, and kanamycin were katG_S315T, rpoB_S450L, gyrA_A90V, and rrs_A1401G, according to observations in North India.^37^ However, the WGS data must be phenotypically and cautiously interpreted with validation software by experts in bioinformatics and compared with reliable database resources.

The isothermal RNA amplification assay is another sensitive tool for detecting drug resistance with little contamination. As the primary target, 85B mRNA of MTB was detected, but the results were often contaminated by residual DNA, and the operation steps were complicated. A study focusing on pre-16S rRNA as the target revealed a sensitivity of 100% and a specificity of 97.4% when rifampicin resistance was detected.^38^

Microarray and high-resolution melt curve analysis (HRM) also explored the detection of drug resistance. The targets of these two methods were rifampicin combined with isoniazid, ethambutol, fluoroquinolones, and second-line injectable drugs. According to Xu *et al*., the sensitivities for judgment of the resistance to INH and RFP by microarray were lower than those detected by Xpert; however, the specificities were comparable.^39^ The benefit of HRM was the extremely high specificities.^40,41^

### How to distinguish latent TB and subclinical TB

For diagnosis of latent TB, two main immune-based approaches are a purified protein derivative (PPD) test and interferon gamma release assay (IGRA)—the two tests have moderate correlation. IGRA operates as follows: blood samples are collected using MTB antigen-coated tubes and interferon-γ release is measured using enzyme immunoassay (EIA) or enzyme linked immunospot (ELISPOT), widely available in countries like the United States or Japan but not available in many developing countries. However, neither test can differentiate latent TB from active TB. Both have poor capacity to predict the activity of latent TB. C-Tb, a skin test based on the *M. tuberculosis*-specific ESAT-6 and CFP10 antigens, has accuracy similar to that of IGRAs in phase 3 clinical trials.^42^

Another disease status called subclinical tuberculosis merits distinction from active TB. Although subclinical TB is active and infectious, patients show no symptoms, whereas in active TB, patients show normal symptoms. In a review, subclinical tuberculosis was described as a disease status with intermittent culture-positive sputum and smear-negative.^43^ Subclinical TB is an intermediate status between latent and active forms of TB, depending on the immune response of the host. The WHO recommended screening persons living with HIV (PLHIV) for active TB by inquiring about four TB-related symptoms: current cough, fever, night sweats, or weight loss. However, the sensitivity of this approach among PLHIV is approximately 79%. This means that some cases of subclinical TB were missed, and such cases could benefit from preventive therapy if diagnosed accurately.^44^ In another study, the prevalence of patients with subclinical TB among PLHIV was 8.5%.^45^ To discriminate them, novel biomarkers need to be explored. During the stage of subclinical TB, active MTB is expected to secrete some metabolites, such as LAM, in the urine or serum. It is hopeful that a more sensitive LAM assay will be developed, which could estimate the risk of individuals progressing to active disease. Fc-mediated antibody (Ab) is another hot topic because of its role in enhancing phagolysosomal maturation and macrophage killing of intracellular MTB via distinct glycosylation patterns compared with Abs from active TB.^46^ In addition, antigen-specific peptides, blood RNA signatures which detect inflammatory signals such as elevated interferon stimulated gene expression,^28,47^ and N-acetylisoputreanine^48^ are promising biomarkers.

### The problem and breakout of monitoring for therapeutic outcomes

Xpert MTB/RIF cannot monitor the effect of TB treatment. Although initiating antibiotic therapy from this test benefits the patient, the test's value for evaluating final outcome is controversial. A meta-analysis was performed to examine improvements in patient outcomes associated with Xpert MTB/RIF.^49^ The authors could not determine whether detection with Xpert MTB/RIF could reduce mortality over a 6-month period among patients, compared with sputum smear microscopy.

SAT was utilized to monitor therapy outcome, which was more sensitive than the acid-fast stain. The therapy feedback could be obtained as early as 2 weeks after chemotherapy started. The positive result of SAT could indicate treatment failure after 5 months without waiting for the results from culture.^50^ The signal intensity of LAM in the urine of six children with TB/HIV became undetectable 3 months after anti-tuberculosis chemotherapy, suggesting that the LAM concentration in urine might be a potential biomarker to monitor the effectiveness of chemotherapy.^51^ Longitudinally detecting the serum concentration of MTB-specific peptide fragments by NanoDisk and high-throughput mass spectrometry also has this effect.^18^

## Strategies for diagnosing TB and prospective strategies for therapeutic monitoring

Passive-case finding is not sufficient to hinder the epidemiology of TB. Novel diagnostic tools are urgently needed. These tools should be clinic-guided and provide a perspective for individualized rather than standardized diagnosis mode. In the past, diagnosis of TB cases followed a standardized strategy, which ignored variation of the host status, disease phase, and mycobacterial molecular character. Here, we propose strategies of personalized medicine-guided diagnosis and therapeutic monitoring that have the significance to drive clinical decision making, based on understanding MTB strains' genome and proteome as well as host immunity and social circumstances.

### Clinical strategy for diagnosing active TB

**Figure 2.**
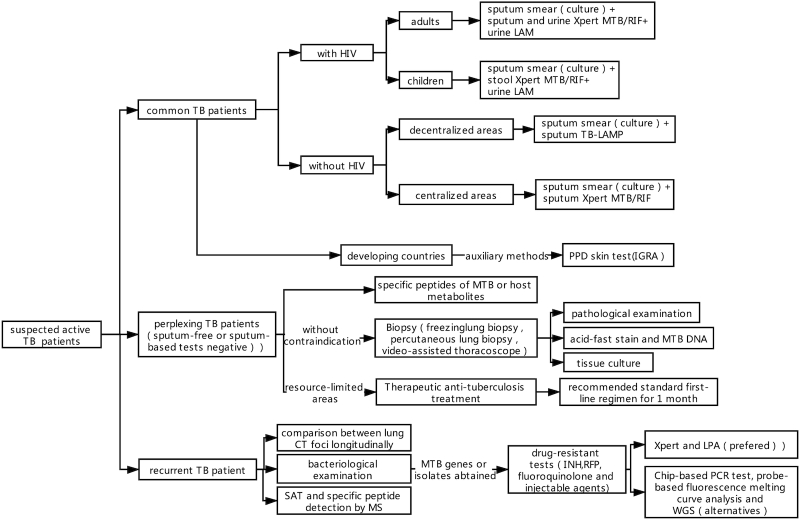
Clinical strategy for diagnosing active TB.

Here, we plot a clinical strategy focusing on different types of subpopulations suffering TB (Fig. 2). When a person suspected of having TB visits a doctor, the doctor should classify him (or her) as follows: a) common TB patient; b) perplexing TB patient; or c) recurrent TB patient.

Common TB patients can be diagnosed under standardized procedures: sputum smear (culture) followed by TB-LAMP in decentralized areas or Xpert in centralized areas. The PPD skin test and IGRA are the auxiliary methods in developing countries. If the patient is infected with HIV, urine LAM should be used. The association between urine LAM and sputum and urine Xpert MTB/RIF in HIV-positive adults as well as between urine LAM and stool Xpert MTB/RIF in HIV-positive children are optimal tactics. Differential diagnosis is an indispensable step.

Perplexing TB patients usually have paucibacillary TB. This population needs to be targeted with a personalized diagnosis strategy. Some patients are sputum-free, and others cannot obtain a final diagnosis after a series of sputum-based tests with negative results. In this situation, there are three possible routes to follow:

- Detection of specific peptides of MTB or host metabolites. To date, the best biomarkers are antigen ESAT-6 and CFP-10. This method is fit for people in different areas of the world.
- Biopsy for pathological examination or etiological detection. New technologies include freezing lung biopsy, percutaneous lung biopsy, and video-assisted thoracoscope. Granuloma observed by pathological section is not a unique characteristic of TB, so acid-fast stain and MTB DNA detection should be undertaken using the obtained tissue.
- Therapeutic anti-tuberculosis treatment. The recommended standard first-line regimen should be taken for 1 month. This method is implemented only in resource-limited areas because of its empiricism essence. Moreover, this approach cannot recognize drug-resistant TB. The diagnosis of recurrent TB has been a clinically difficult problem for a long time. In addition to sputum bacteriological examination and comparison between lung CT foci longitudinally, SAT and specific peptide detection by MS are recommended.

Detection of drug-resistant TB is essential and is proposed to start once the MTB genes or isolates have been obtained, especially in patients with high risk factors for drug-resistant TB. Resistance for not only INH and RFP but also fluoroquinolone and injectable agents must be considered. Xpert and LPA are preferable tests, and the Chip-based PCR test, probe-based fluorescence melting curve analysis, and WGS are alternatives.

### Strategy for diagnosing LTBI and subclinical TB

In high-prevalence countries, TST is the major screening test. TST(++) or (+++) indicate LTBI. IGRA is used when the subject is immune-compromised or it is difficult to distinguish BCG-vaccinated individuals from individuals with LTBI. In low-prevalence countries (usually developed countries), IGRA is applied to directly screen for LTBI. The C-Tb skin test is another choice if the price of IGRA is not acceptable. The definition of subclinical TB is still obscure, and clarifying this definition is an urgent issue. LAM is a hopeful biomarker for diagnosing this type of TB.

### Strategy for monitoring therapeutic outcomes

Similar to the issue of recurrent TB, we must find a way to detect the existence of active MTB and estimate the amount or decline of the bacterial load. The role of SAT and the detection of LAM and ESAT-6 need to be validated in large populations in the future, and, hopefully, they can indicate a therapeutic effect in less than 2 weeks.

## Future directions and conclusion

In the future, it is hoped that promising molecular technologies for active TB including Xpert, WGS, and NanoDisk-MS yield brilliant results. Xpert should be perfected so that it can be more sensitive and be applied for detecting resistance to more drugs (especially the key drugs belonging to the A group on the 2018 WHO guidelines). However, we must interpret the results cautiously because of the low positive predictive value in the low epidemic countries and areas. For WGS, the challenges include the following: low nucleic acid yield when sequencing directly from sputum, high cost, bioinformatics-based analysis workflow, and discordance in phenotypic and genotypic drug resistance data. Hopefully, developed technologies for enrichment TB DNA signal, generalization of targeted NGS ($50-$100 per sample), application of knowledge-based and user-friendly databases e.g. ReSeqTB, PhyResSE would be effective solutions. On the other hand, the relationship between the phenotype and genotype of drug resistance needs to be explored more deeply. Bioinformaticians, molecular biologists, and clinical doctors need to engage together more closely. NanoDisk-MS can be carried out to discover more specific antigens of MTB and more host metabolites, and the strategy of identification for a combination of compounds can be developed. A validation test of these biosignatures must be emphasized. The correlation between drug resistance and specific peptides is worth determining. The technologies mentioned above also can be combined with imaging techniques, and even artificial intelligence, so that we can obtain more accurate results.

More immune molecules or immune methods can be used to determine the patient diagnosis of LTBI and subclinical TB. Biomarkers screened should reflect early host-pathogen interactions, concerning bacterial load (bacterial components in body fluids, antibodies, cytokines concerned about innate immune response and MTB-specific T cell responses), early pathology (metabolic or proteomic disturbances related to lung pathological abnormality), and novel imaging modalities.

Biomarkers for monitoring therapeutic outcomes still need to be discovered. It must be clarified how disease outcomes are associated with the status of the bacteria and the host. Not only biosignatures indicating bacterial load, but also host biomarkers reflecting immune response, organ function, quality of life, and expected lifespan should be taken into consideration.

In summary, new diagnostic tools for TB are robust, and nucleic acids, proteins, and metabolites can all be screened. We can detect them to diagnose different phases of TB, determine whether MTB is drug-resistant, and evaluate therapeutic outcomes. A standard diagnosis procedure is not enough, however, and a personalized diagnosis strategy is preferable.
